# Relationships between organizational support, emotional intelligence and work engagement among Chinese nurses: A correlation study

**DOI:** 10.1002/nop2.70034

**Published:** 2024-10-15

**Authors:** Xing Gao, Yanli Zhou, Xiao Xu, Ran Yuan, Yanxue Zheng, Ren Yun

**Affiliations:** ^1^ Gynecology Comprehensive Ward of Affiliated Hospital of Jining Medical University Jining Shandong China; ^2^ Gynecology Ward 1 of Affiliated Hospital of Jining Medical University Jining Shandong China; ^3^ Gynecology Ward 3 of Affiliated Hospital of Jining Medical University Jining Shandong China; ^4^ Anesthesiology of Affiliated Hospital of Jining Medical University Jining Shandong China; ^5^ Gynecology Ward 2 of Affiliated Hospital of Jining Medical University Jining Shandong China

**Keywords:** emotional intelligence, job engagement, mediating effect, nurses, sense of organizational support

## Abstract

**Aim:**

To investigate and analyse the current situation of clinical nurses' sense of organisational support, emotional intelligence and work engagement in different departments and their correlation, in order to provide guidance for enhancing the sense of organisational support, improving nurses' emotional intelligence and mobilising nurses' work engagement and to promote the development of nursing teams.

**Methods:**

Clinical nurses from three hospitals in eastern and western China were selected by convenience sampling method from September to October 2023 and were surveyed using a general information questionnaire, organisational support questionnaire, emotional intelligence scale and work engagement scale. We investigated the correlation between nurses' sense of organisational support, emotional intelligence and work engagement in clinical units.

**Results:**

The clinical nurses investigated in this study had moderate levels of perceived organisational support and emotional intelligence and high levels of work engagement. The total score of the nurses' sense of organisational support questionnaire was positively correlated with the total score of the emotional intelligence scale, the total score of the work engagement scale and the scores of all dimensions (all *p* < 0.05). Emotional intelligence played a partial mediating effect in the effect of nurses' organisational support on work engagement, accounting for 35.57% of the total effect.

**Conclusion:**

Clinical nurses' sense of organisational support was positively correlated with emotional intelligence and work engagement; there was a chain mediating effect of clinical nurses' emotional intelligence between sense of organisational support and work engagement. Organisational support can enhance the emotional intelligence of caregivers as well as promote work engagement. It is suggested that nursing managers can enhance the level of nurses' engagement by giving effective organisational support and at the same time improving nurses' perception of psychological safety.

## 1 INTRODUCTION

At the beginning of the 21st century, the American Nurses Credentialing Center Magnetic Nursing Conference (American Nurses Credentialing Center, ANCC) clearly pointed out that nurses are playing an increasingly important role in the healthcare industry (Qingguang et al., 2022). China's ‘Healthy China 2030’ outline also puts forward the ‘big health, big health’ concept of all‐round, whole‐life cycle maintenance of people's health, nurses as the construction of the main force, in the optimization of the health service system, to promote healthy lifestyles. As the main force of construction, nurses have an irreplaceable role in optimizing the health service system, promoting healthy lifestyles, facilitating early recovery of patients and preventing recurrence of diseases. However, at the same time, we are also facing the challenges of a relatively insufficient number of nurses and a high departure rate. The development of the nursing career is still unbalanced and insufficient (Howard, 2021). Based on the above problems, China's National Health Commission coordinated reforms and released the National Nursing Career Development Plan (2021–2025) in 2022, which explicitly proposed to strengthen the nursing workforce to fill the gap in nurses' positions with the theme of high‐quality development and focused on the cultivation of talents in tertiary hospitals (Journal News, 2022). In summary, how to mobilize the enthusiasm of the majority of nurses and promote the development of the nursing team has become a hot spot in the current research.

The concept of work engagement has attracted a great deal of attention from scholars in the psychological community since it was first introduced in Kahn (1990) and Schaufeli et al. (2002) interpreted it as the degree of energy, focus and dedication that an employee exhibits at work and developed the UWES (Utrecht Work Engagement Scale), which is now a clinically recognized and popularized scale, which is now clinically recognized and promoted. Although work engagement makes a significant contribution to organizational development, the current work engagement of clinical nurses needs to be strengthened (Heyns & Rothmann, 2021). Nurses who are committed, enthusiastic and dedicated to their work are considered to be highly engaged in their work, which can lead to continuous improvement in quality and patient satisfaction and increase the influence and reputation of the hospital.

The concept of Perceived Organizational Support (POS) was proposed by Eisenberger et al. (1986) and initially applied to management science and attracted widespread attention (Chen, 2019). When employees have a high sense of organizational support, their performance will increase, while low organizational support is closely related to turnover intention (Zhe & Jianhong, 2021). In the traditional hospital management system, the role played by the leadership is mostly that of a manager rather than a supporter, and nurses are also given the role of devotees, coupled with the high intensity and heavy task of nursing work, the nurse group is considered to be a high‐stress group of people, which shows that the problem of the lack of organizational support for the clinical nursing staff needs to be paid attention to.

Salovey and Mayer (1990) proposed the framework of emotional intelligence at the end of the 20th century, defining the concept as the basic skill of accurately assessing and expressing the emotions of others and oneself, and Caldwell (2021) mentioned that ‘emotional intelligence’ plays a more important role than ‘emotional intelligence’ in social adaptation. ‘Emotional intelligence’ plays a higher role than IQ in social adaptation and emotional intelligence plays a crucial role in practice, career development and management. Currently, nurses facing heavy work pressure will produce physical and mental fatigue, affecting the emotional state at work, so improving emotional intelligence can better cope with work pressure, reduce the physical and mental fatigue brought about by work, improve occupational well‐being and improve the effectiveness of nursing work (Juan, 2020; Weihua, 2021). Relevant studies have shown that clinical nurses show a strong interest in improving their emotional intelligence (Lu & Shorey, 2021), so this study suggests that emotional intelligence should be incorporated into nursing curricula and emphasized by administrators.

Most of the studies that have been conducted have investigated the current status of organizational support or emotional intelligence of nurses in general clinical units, with relatively few studies on the correlation between the variables and whether or not they interact with each other, in addition to very few studies that have applied the Job Demand Resource Model to the field of organizational behaviour. Using the work demand resource model as the basic framework and the Emotion Regulation Theory and Conservation of Resources Theory, this study investigates the current status of clinical nurses' sense of organizational support, current status of emotional intelligence and current status of work engagement and clarifies the factors affecting the differences in each variable and explores the mechanism of the variables, so as to guide nurses to promote the high‐quality development of the nursing team in clinical practice. We investigated the current status of clinical nurses' organizational support, emotional intelligence and work engagement, identified the influencing factors of the differences in each variable and explored the mechanism of the variables, in order to guide the nurses' work engagement in clinical practice and to promote the high‐quality development of the nursing team.

## 2 METHODS

### 2.1 Participants of the survey

Adopting the convenience sampling method, which is the simplest and most convenient sampling method, allows the researcher to collect sample data more easily as individuals are selected based on their availability and willingness to participate. Based on the descriptive research sample size formula: sample size = (number of variables) × (5–10) × (1 + [10%–15%]); there were 21 variables in this survey, and considering 20% invalid questionnaires, the final sample size was determined to be 252–504. The sample size of this study was 258.

Inclusion criteria: (1) obtained a certificate of nursing practice; (2) participated in a national nursing skills competition; (3) informed consent and voluntary participation.

The exclusion criteria were (1) nurses who were studying in outside hospitals and (2) nurses who were not on duty during the survey period, such as leave of absence from the hospital, going out for further study or training and so on. The study participants were informed statistically and voluntarily participated in this study.

### 2.2 Measurements

#### 2.2.1 General Information Questionnaire

The general demographic information questionnaire of this study incorporated a total of 11 variables of general demographic and work‐related information. The basic information included gender, age, marital status and education and work‐related information included department, title, years of service, grade, number of night shifts per month, nature of employment, salary status and type of hospital.

#### 2.2.2 Sense of Organizational Support Scale

Perceived Organizational Support Questionnaire (POSQ) was developed using the Perceived Organizational Support Scale (POSS) developed by Chen (2006) based on Eisenberger's definition of POS, with reference to Mcmillin's Instrumental Support (IAS) and the Exploratory Factor Analysis (EFA) of Ling Wen wheeling on domestic employees. It contains two dimensions of emotional support and instrumental support, with a total of 13 entries. A five‐point Likert scale was used, with 1 being ‘very inconsistent’ and 5 being ‘very consistent’. The higher the score, the higher the actual support nurses feel in the hospital. The scale has good reliability and validity, and the Cronbach's α coefficient of the questionnaire in this study was 0.987 and the Cronbach's α coefficients of the dimensions were 0.963–0.985.

#### 2.2.3 Emotional Intelligence Scale

The scale was compiled from Wong and Lawl's English version (Wong & Law, 2002). Each of the four entries of the scale is a section, in order of emotion perception, integration, utilization and comprehension, and is scored on a five‐point Likert scale of 1 (extremely non‐compliant) to 5 (extremely compliant). The total score ranges from 16 to 80. The Cronbach's *α* coefficient for this scale in this study was 0.940 (>0.8).

#### 2.2.4 Work Input Scale

The scale was translated from the English version of Schaufeli's (Binghai et al., 2014) by our scholar Yi‐Wen Zhang. The scale contains nine items, which are categorized into vitality dimension, dedication dimension and concentration dimension. A seven‐point scale from 0 (never) to 6 (always) was used. The Cronbach's *α* coefficient of this scale in this study was 0.947 (>0.8) with good validity.

### 2.3 Data collection methods

This study utilized the Questionnaire Star questionnaire for data collection. The edited questionnaire was filled into the Questionnaire Star system by the members of the research team on September 2023 and checked by two people for accuracy. The questionnaire was distributed to the microblogging group with the consent of the authorities. The first page of the questionnaire was set up with a unified guidance instruction language, informing the purpose of this survey, the method and the problems to be noted in the process of filling out, etc. This study followed the principle of voluntariness. At the end of the survey, two researchers verified the questionnaires submitted by the respondents one by one, and excluded the questionnaires that did not meet the requirements in time. Finally, 258 valid electronic questionnaires were obtained.

### 2.4 Statistical methods

It was analysed using SPSS 26.0 and AMOS 21.0 statistical software. General information was analysed by descriptive statistics, count data were described by frequency and percentage and measurement data were described by mean ± standard deviation; correlations between nurses' work input and variables were analysed by Pearson correlation analysis; and mediation effects were analysed by AMOS 21.0 software. Differences were considered statistically significant at *p* < 0.05.

## 3 RESULTS

### 3.1 General information on clinical nurses

A total of 258 study participants were included in this study, 20 (7.8%) were male and 238 (92.2%) were female; 22 (8.5%) had college education or less, 236 (91.5%) had bachelor's degree or above; 55 (21.3%) were in non‐marital status and 203 (78.7%) were in marital status; titles: 27 (10.5%) were nurses, 74 (10.5%) were nurse practitioners (28.7%), 120 (46.5%) supervisory nurse, 37 (14.3%) associate nurse and above; other general information is shown in Table 1.

**TABLE 1:** General demographic characteristics of clinical nurses (*N* = 258).

Variant	Categorization	Number of persons	Proportion (%)
Distinguishing between the sexes	Male	20	7.8
	Women	238	92.2
(A person's) Age	18–25 years	28	10.9
	26–30 years	45	17.4
	31–35 years	79	30.6
	36–40 years	49	19
	41 and over	57	22.1
Marital status	Married	203	78.7
	Unmarried	49	19
	Divorced or widowed	6	2.3
Title	Physiotherapists	27	10.5
	Physiotherapists	74	28.7
	Nurse practitioner‐in‐charge	120	46.5
	Associate nurse practitioner and above	37	14.3
Years of experience	Less than 5 years	53	20.5
	6–10 years	50	19.4
	11–15 years	71	27.5
	16–20 years	47	18.2
	21 years and over	37	14.3
Education attainment	Specialized and below	22	8.5
	Undergraduate (adjective)	221	85.7
	Graduate students and above	15	5.8
Level of hospital	First‐class	3	1.2
	Category B	29	11.2
	Three‐tier	226	87.6
Number of night shifts per month	<6 days	153	59.3
	>6 days	105	40.7
Nature of employment	Authorized strength	65	25.2
	Contract employment	136	52.7
	Personnel agent	57	22.1
Payroll	<3000	2	0.8
	3000–5000	53	20.5
	5000–10,000	138	53.5
	10,000–15,000	44	17.1
	>15,000	21	8.1
Whether you have participated in a national skills competition	Be	10	3.9
	Clogged	248	96.1
Administrative division	Nursing department	2	0.8
	Emergency department	30	11.6
	Custody room	30	11.6
	General medicine	45	17.4
	Operating rooms	28	10.9
	Neurosurgery	123	47.7

### 3.2 Clinical nurses' scores on the Sense of Organizational Support Questionnaire, Emotional Intelligence Scale and Work Engagement Scale

The Organizational Support, Emotional Intelligence and Work Engagement scales and their individual dimension scores approximately follow a normal distribution and are expressed as mean ± standard deviation. In total, 258 nurses scored (45.20 ± 9.91) on the Work Engagement Scale and the mean scores for each dimension entry were, in descending order, VIGOUR (5.22 ± 1.18), dedication (5.00 ± 1.16), concentration (4.84 ± 1.16) score. The Emotional Intelligence Scale score was (61 ± 8.97) and the mean scores of the dimensional entries from highest to lowest were emotion perception (3.95 ± 0.68), emotion utilization (3.80 ± 0.69), emotion understanding (3.76 ± 0.69) and emotion integration (3.73 ± 0.70). The total score of Organizational Sense of Support Questionnaire was (46.56 ± 10.08) and the score of Instrumental Support Dimension (4.84 ± 1.16) was lower than the score of Emotional Support Dimension (5.11 ± 1.14), as shown in Table 2.

**TABLE 2:** Clinical nurses' work engagement, emotional intelligence and organizational support scores (*N* = 258).

Variant	Sports event	Entry	Scoring range	Score	Entry parity (accountancy)
Devote one's energies to work		9	0–54	45.20 ± 9.91	5.02 ± 1.10
	Vigour	3	0–18	15.68 ± 3.53	5.22 ± 1.18
	Dedication	3	0–18	14.99 ± 3.47	5.00 ± 1.16
	Single‐mindedly devoted to	3	0–18	14.52 ± 3.47	4.84 ± 1.16
Emotional intelligence		16	16–80	61 ± 8.97	3.81 ± 0.56
	Emotional perception	4	4–20	15.81 ± 2.71	3.95 ± 0.68
	Emotional use	4	4–20	15.21 ± 2.78	3.80 ± 0.69
	Emotional understanding	4	4–20	15.05 ± 2.77	3.76 ± 0.69
	Emotional integration	4	4–20	14.92 ± 2.79	3.73 ± 0.70
Organizational support		13	13–65	46.56 ± 10.08	3.58 ± 0.78
	Emotional support	10	10–50	36.09 ± 7.69	5.11 ± 1.14
	Instrumental support	3	3–15	10.47 ± 2.64	4.84 ± 1.16

### 3.3 Correlation analysis of clinical nurses' sense of organizational support, emotional intelligence and work engagement

The total score of the Nurses' Sense of Organizational Support Questionnaire was positively correlated with the total score of the Emotional Intelligence Scale, the total score of the Work Engagement Scale and the scores of all dimensions (all *p* < 0.05). See Table 3 for details.

**TABLE 3:** Correlation between clinical nurses' work engagement, emotional intelligence and organizational support (*r*).

	Single‐mindedly devoted to	Dedication	Vigour	Total work input score	Emotional perception	Emotional integration	Emotional use	Emotional understanding	Total emotional intelligence score	Instrumental organizational support	Emotional organizational support	Total organizational support score
Single‐mindedly devoted to	1											
Dedication	0.843^a^	1										
Vigour	0.798^a^	0.894^a^	1									
Total work input score	0.929^a^	0.963^a^	0.948^a^	1								
Emotional perception	0.440^a^	0.470^a^	0.435^a^	0.473^a^	1							
Emotional integration	0.509^a^	0.516^a^	0.447^a^	0.518^a^	0.493^a^	1						
Emotional use	0.566^a^	0.612^a^	0.528^a^	0.600^a^	0.541^a^	0.608^a^	1					
Emotional understanding	0.419^a^	0.446^a^	0.381^a^	0.438^a^	0.487^a^	0.537^a^	0.611^a^	1				
Total emotional intelligence score	0.595^a^	0.629^a^	0.551^a^	0.625^a^	0.773^a^	0.814^a^	0.851^a^	0.812^a^	1			
Instrumental organizational support	0.545^a^	0.619^a^	0.547^a^	0.602^a^	0.421^a^	0.453^a^	0.517^a^	0.426^a^	0.560^a^	1		
Emotional organizational support	0.534^a^	0.637^a^	0.585^a^	0.618^a^	0.426^a^	0.513^a^	0.478^a^	0.372^a^	0.551^a^	0.871^a^	1	
Total organizational support score	0.551^a^	0.648^a^	0.590^a^	0.630^a^	0.436^a^	0.511^a^	0.501^a^	0.395^a^	0.567^a^	0.927^a^	0.992^a^	1

^a^ Significantly correlated at the 0.01 level (bilaterally).

### 3.4 The mediating role of clinical Nurses' sense of organizational support, emotional intelligence and work engagement

Model 4 in the SPSS add‐in process was used to test the mediation effect after controlling the general information with the organizational support of nurses as the independent variable, work engagement as the dependent variable and emotional intelligence as the mediator variable. Bootstrap sample size value was set at 5000 with 95% confidence interval to get the results of data analysis.

From the analysis results in Table 4, organizational support was able to significantly and positively predict work engagement (*β* = 0.62, *t* = 12.97, *p* < 0.001) and the fitting index *R* ^2^ showed that the predictive model of organizational support on work engagement explained 39.6% of the variance. When organizational support and emotional intelligence predicted work engagement simultaneously, nurses' organizational support significantly and positively predicted work engagement (*β* = 0.40, *t* = 74.20, *p* < 0.001), while emotional intelligence significantly and positively predicted work engagement (*β* = 0.44, *t* = 12.81, *p* < 0.001). The fit index *R* ^2^ showed that the predictive model of organizational support and emotional intelligence on work engagement explained 39% of the variance.

**TABLE 4:** Analysis of the mediating effect of emotional intelligence on the relationship between organizational support and work engagement.

Outcome variable	Predictor variable	Estimated coefficient	Fitness index		Significance of the coefficient	
			*R* ^2^ value	*F*‐value	*t*‐value	*p*‐value
Devote one's energies to work	Organizational support	0.6193	0.396	168.07	12.966	<0.001
Emotional intelligence	Organizational support	0.5047	0.319	121.463	11.021	<0.001
Devote one's energies to work	Emotional intelligence	0.4364	0.390	164.101	12.807	<0.001
	Organizational support	0.3990	–	–	74.204	<0.001

**FIGURE 1:**
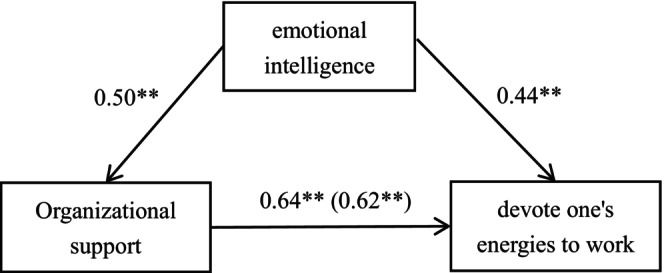
Model of the mediating effect of emotional intelligence on the relationship between organizational support and work engagement. 0.50** and 0.44** are mediating effects of the mediating variable Q; 0.64** (0.62**) is a direct effect; **p < 0.01.

As can be seen from the results of the analysis in Table 5, when the confidence interval is 95%, the mediator test result for self‐regulation fatigue does not include 0 (LLCI = 0.13, ULCI = 0.33), which indicates that emotional intelligence plays a partially mediating effect in the effect of organizational support on work engagement in nurses, which accounts for 35.57% of the total effect. Therefore, a diagram of the model of clinical nurses' organizational support emotional intelligence one work engagement can be derived and is shown in Figure 1.

**TABLE 5:** Total, direct and indirect effects of emotional intelligence on the relationship between organizational support and work engagement.

Sports event	Efficiency value	Boot SE	95% confidence interval		Relative affect value (%)
			Limit	Lower limit	
Aggregate effect	0.6193	0.0478	0.5252	0.7133	–
Direct effect	0.3990	0.0528	0.2951	0.5029	0.6443
Indirect effect	0.2203	0.0512	0.1267	0.3267	0.3557

*Note*: Upper and lower bounds of standard errors, 95% confidence intervals for indirect effects estimated using the Bootstrap method.

## 4 DISCUSSION

### 4.1 Analysis of the current status of clinical nurses' organizational support, emotional intelligence and work engagement

In this study, the actual score for the sense of organizational support was (46.56 ± 10.08) and the total entry mean score was (3.58 ± 0.78), which is still in the middle of the total mean score for the sense of organizational support in relation to the range of mean score scores from 1 to 5. For nursing staff the instrumental support dimension scores were lower than the emotional support dimension scores, a result that differs from previous results (Jinjin, 2019; Zhaonan, n.d.). This result suggests that in recent years, hospital management has been increasingly focusing on the emotional needs of the nursing community from a human‐centred perspective, paying attention to the value and physical and mental health of nurses, so that they can develop a sense of belonging to the organization, enhance their professional identity and professional emotions, which in turn improves their affective domain competence, which in turn is used as a kind of internal incentive to the organization, which in turn improves the quality of their services and provides them more efficiently.

Emotional intelligence was at a moderate level (3.81 ± 0.56), similar to previous studies (Xiao et al., 2021), the dimension with the highest score among the dimensions was self‐emotional assessment (3.95 ± 0.68) and the dimension with the lowest score was emotional management (3.73 ± 0.70). The dimension of self‐emotional assessment scored the highest, which is consistent with the findings of Zhang et al. (2010). It may be due to the fact that the respondents of this survey are mainly female, accounting for 90.6%, and females are relatively delicate and are more able to clearly understand their inner feelings and understand their emotional changes. Emotional utilization dimension is higher, because nurses' work environment is facing vulnerable groups, need higher emotional intelligence to serve patients and their families, frequent interpersonal communication with patients, reduce the pressure on nurse–patient communication and accumulate more emotional experience. Emotional management scores were lower for the following possible reasons: nurses are burdened with the responsibility of maintaining and promoting patients' health, the diversity of the population they serve, high occupational stress and prolonged night shifts, which leads to self‐doubt, enhanced emotional exhaustion and a tendency to have poorer emotional control.

Work engagement is a positive and satisfying emotional and cognitive state related to work that is enduring and diffuse. The results of this study showed that the nurses' work engagement score was (45.20 ± 9.91) and the mean of the entries was (5.02 ± 1.10) and according to the division criteria of Li‐Min et al. (2020), 0–2 was at a low level, 2–4 was at an intermediate level and ≧4 was at a high level, which indicated that the nurses' work engagement was at a high level. In this study, nurses had the highest score of vitality dimension (5.22 ± 1.18) and the lowest score of concentration dimension (4.84 ± 1.16), which is consistent with the results of Zanmei et al. (2019) and is related to the fact that most of the personnel who participated in the survey of this study were under 35 years old and were in the middle of the road, and it also indicates that clinical nurses have high motivation to devote themselves to their work even though their work content is more demanding. Clinical nurses are required to have higher professional quality and more rigorous work attitude, but the reality is that the relative shortage of nursing human resources leads to an increase in workload, coupled with long working hours, distrust of patients' families and tensions in the relationship between doctors and nurses, thus affecting their focus. Therefore, the nursing management level should pay attention to the work commitment of clinical nurses and give corresponding support measures, such as increasing manpower, flexible scheduling and setting up dedicated staff to assist the work of responsible nurses, so as to enhance the level of their commitment to their work, so as to make further improvements and mobilize the enthusiasm of nurses.

### 4.2 Correlation analysis of clinical nurses' organizational support, emotional intelligence and work engagement

Pearson correlation analysis showed a positive correlation between sense of organizational support and work engagement (*r* = 0.630, *p* < 0.001), between sense of organizational support and emotional intelligence (*r* = 0.567, *p* < 0.001) and between emotional intelligence and work engagement (*r* = 0.625, *p* < 0.001). The above results are consistent with the results of the studies conducted by Luo et al. (2021), Ting et al. (2015) and Zengjian and Qing (2020), respectively, indicating that the three variables of clinical nurses' sense of organizational support, emotional intelligence and work engagement are correlated with each other and mutually influence each other.

The results of this study show that a higher sense of organizational support can effectively and quickly alleviate the emotional stress brought about by various aspects, which is conducive to the cultivation of emotional intelligence, in addition to strengthening the nurses' sense of ownership, enhancing the sense of security so that nurses can treat their work with a higher degree of initiative and maintain an enthusiastic commitment to their work. Hospitals should actively create resources for clinical nurses to increase the sense of organizational support, such as the provision of relevant equipment for the department, for nurses to fight for training opportunities to protect the rights and interests of nurses and at the same time to be humanistic care to enhance the sense of belonging to the nurses, pay attention to the demands of the staff to provide emotional support, focusing on the nurses to pay attention to the expression of the value of identity, all‐round to improve the nurses for the perception of emotions, integration, application and understanding of the level of alleviate the fatigue brought about by the clinical nursing. It also improves the level of nurses' perception, integration, utilization and understanding of emotions, alleviates the fatigue brought by clinical nursing and improves work motivation and concentration. At the same time, there is a positive correlation between emotional intelligence and work commitment, that is, the higher the emotional intelligence of nurses, the higher the work commitment, so nurses need to strengthen the cultivation of emotional intelligence, improve the ability to manage emotions, learn to think differently and adjust themselves to maintain a positive work attitude when they are emotionally depressed or overly excited and when they cannot self‐digest they should be able to use the resources around them to reduce the emotional exhaustion and promote work commitment. Promote work engagement.

### 4.3 Analysis of the mediating effects of organizational support, emotional intelligence and work engagement among clinical nurses

There is a two‐by‐two significant correlation between organizational support, emotional intelligence and work engagement among clinical nurses, which is suitable for mediation effect analysis. This study showed that the direct effect of organizational support on work engagement was 0.6193 and emotional intelligence partially contributed to the relationship between organizational support and work engagement with a mediated effect estimate of 0.3990 (*p* < 0.001).

On the one hand, according to the social exchange theory, when clinical nurses perceive more organizational support, they will work harder in return, so as to maintain their enthusiasm and increase their work commitment; on the other hand, the sense of organizational support will create a good working atmosphere for the clinical nurses, which will make the nurses full of psychological security and thus more courageous to take the initiative in their work (Aldabbas et al., 2023). This also confirms the direct positive effect of clinical nurses' sense of organizational support on their work engagement in this study.

This study found that nurses' emotional intelligence had a significant moderating effect between organizational support and work engagement. This is shown by the fact that nurses with a strong sense of organizational support have a stronger effect of organizational support on their work engagement as their emotional intelligence increases. In other words, nurses with a high level of overall emotional intelligence are more likely to increase their individual work engagement and have a high level of work engagement; nurses with a lower level of emotional intelligence are not conducive to work engagement and have a low level of work engagement. Emotional regulation refers to the process by which an individual is able to adjust his or her emotions, to realize the causes of emotional changes in the face of external disturbances that cause emotional changes and to adjust them so as to cause corresponding changes in subjective responses and coping with stress (Gross & John, 2003). Nurses with low emotional intelligence cannot perceive their own emotional changes in time, it is difficult to regulate their own emotional problems in time, it is more difficult to understand the emotions of others, it is not conducive to the regulation with the surrounding environment, it is easier to produce negative emotions and it is impossible to complete the clinical work successfully, thus reducing the degree of work commitment (Jaracz et al., 2017). Therefore, it is crucial for nursing managers to increase the cultivation of nurses' emotional intelligence and improve their emotional intelligence.

Other studies (Dingxin et al., 2021; Jun et al., 2020; Luo et al., 2021; Shihan et al., 2020) have shown that in addition to the direct effects described above, clinical nurses' sense of organizational support can also have an indirect effect on work engagement through other variables. The higher the clinical nurses' sense of organizational support, the better the working environment for nurses in the hospital and the higher the nurses' emotional intelligence (Xiao et al., 2021). According to the theory of emotional regulation, reasonable emotional regulation can reduce negative emotional experiences, stimulate work motivation and have a positive impact on work engagement (Emelia, 2022). In addition, nurses with good organizational support can maintain positive emotions towards their work (Feng, 2019). They have a stronger sense of responsibility to achieve their work goals more firmly and they will be more focused in their work. In addition, nurses with good organizational support can maintain positive emotions about their work, have a stronger sense of responsibility to achieve their work goals more firmly and devote themselves to their work in a more focused state. In conclusion, the mediating effect of organizational support–emotional intelligence–work engagement was established in this study.

## 5 INNOVATIVENESS

First, this study explored the relevant role mechanisms among clinical nurses' organizational support, emotional intelligence and work engagement from the personal resources in social exchange theory, expanding new theories and bases for the study of nurses' organizational support, emotional intelligence and work engagement.

Secondly, in terms of content, the correlation analysis of clinical nurses' emotional intelligence in organizational support and work engagement was conducted quantitatively, which verified the mediating effect of clinical nurses' emotional intelligence between organizational support and work engagement and enriched the related research content to a certain extent.

## 6 LIMITATIONS

The study questionnaire only involved clinical nurses in some of the tertiary general hospitals in China, and the proportion of female participants was high, while the proportion of male participants was relatively low, which resulted in a selective bias of the sample. It is recommended that follow‐up studies should be designed to minimize sample bias and further increase the credibility of the findings. In addition, this study could not explain the causal relationship between variables based on cross‐sectional surveys and longitudinal cohort studies and intervention studies are still needed to confirm the findings of this study.

## 7 CONCLUSION

This study clarified the current status of Chinese nurses' perceptions of organizational support, emotional intelligence and work engagement and constructed a relationship model among the three variables, confirming the direct predictive effect of perceptions of organizational support on work engagement and the indirect predictive effect of perceptions of organizational support on work engagement through emotional intelligence and exploring the mechanism of interactions among the variables to provide a basis for the development of future interventions.

The role of emotional intelligence in the relationship between organizational support and work engagement provides a new direction for clinical nursing managers and researchers, who can enhance the level of nurses' dedication by giving effective organizational support while improving nurses' perceptions of psychological safety.

## AUTHOR CONTRIBUTIONS

X.G. and Y.Z. acquired the data and designed the study. X.G. and Y.X.Z. performed the statistical analysis and made major contributions to the conception and design of the study and data acquisition, analysis and interpretation. X.X. prepared the draft of the manuscript. Ra.Y., R.Y. and X.X. also revised this manuscript. All authors read and approved the final manuscript.

## FUNDING INFORMATION

There is no funding for the study.

## CONFLICT OF INTEREST STATEMENT

The authors declare no conflicts of interest.

## ETHICS STATEMENT

The Committee of Research Ethics of Affiliated Hospital of Jining Medical University (decision date: 2023‐10‐C012) reviewed the ethical aspects of the research project and issued a statement regarding its ethical acceptability. We conducted three anonymous, self‐administered questionnaires and considered answering the questionnaire as consent to participate in the study.

## Data Availability

All data supporting our findings were presented within the manuscript.
